# The relationship between internet gaming disorder and psychotic experiences: cyberbullying and insomnia severity as mediators

**DOI:** 10.1186/s12888-023-05363-x

**Published:** 2023-11-17

**Authors:** Feten Fekih-Romdhane, Eya Lamloum, Alexandre Andrade Loch, Wissal Cherif, Majda Cheour, Souheil Hallit

**Affiliations:** 1 The Tunisian Center of Early Intervention in Psychosis, Department of Psychiatry “Ibn Omrane”, Razi hospital, Manouba, 2010 Tunisia; 2 Faculty of Medicine of Tunis, Tunis El Manar University, Tunis, Tunisia; 3 Laboratorio de Neurociencias (LIM 27), Instituto de Psiquiatria, Hospital das Clinicas HCFMUSP, Faculdade de Medicina, Universidade de Sao Paulo, Sao Paulo, SP, BR Brazil; 4 Instituto Nacional de Biomarcadores em Neuropsiquiatria (INBION), Conselho Nacional de Desenvolvimento Cientifico e Tecnológico, Sao Paulo, Brazil; 5 School of Medicine and Medical Sciences, Holy Spirit University of Kaslik, P.O. Box 446, Jounieh, Lebanon; 6 Research Department, Psychiatric Hospital of the Cross, Jal Eddib, Lebanon; 7 Applied Science Research Center, Applied Science Private University, Amman, Jordan

**Keywords:** Internet gaming disorder, Psychotic experiences, Cyberbullying, Insomnia, Students, Psychosis risk

## Abstract

**Background:**

The nature of the relationship between Internet Gaming Disorder (IGD) and psychosis is unclear so far. There is evidence that greater time spent in playing video games may expose players to both insomnia and a toxic online environment with widespread cyberbullying. These two possible consequences of IGD may, in turn, be associated with greater psychotic experiences (PE). Based on this theoretical framework, the present study proposed to contribute the body of the knowledge in this area, by testing the possible indirect effects of insomnia severity, cyber-victimization and cyberbullying in the cross-sectional association between IGD and PE in a sample of Tunisian university students.

**Method:**

We conducted a cross-sectional study over 4 months (February-May 2022). The Arabic versions of the Brief Symptom Inventory, the Internet Gaming disorder-20 Test, the Insomnia Severity Index, and the Revised Cyber Bullying Inventory–II were administered to a total of 851 students (mean age = 21.26 ± 1.68 years, 53.7% females).

**Results:**

We found that 25% of students were at risk of IGD, and 1.8% had an IGD. The results of the mediation analysis showed that insomnia severity fully mediated the association between IGD and paranoid ideation. Higher IGD was significantly associated with more insomnia severity, which was, in turn, significantly associated with more paranoid ideation. Cyberbullying partly mediated the association between IGD and psychoticism. Higher IGD scores were significantly associated with more cyberbullying, which was, in turn, significantly associated with more psychoticism. Finally, greater IGD was significantly and directly associated with higher psychoticism.

**Conclusion:**

Our findings suggest that insomnia and cyberbullying may be regarded as potential targets for youth mental health promotion, as well as community-focused prevention and early intervention in psychosis. More particular attention should be devoted to the huge potential for engaging in cyberbullying among online gamers. Sleep deprivation should be prevented, assessed and treated in heavy gamers.

## Background

Online gaming has currently become one of the preferred sources of recreation and communication for youth [1], and this tendency has increased during the COVID-19 and negatively impacted their mental well-being [2]. A systematic review and meta-analysis encompassing 53 studies and 226,247 participants from 17 countries found an estimated worldwide-pooled prevalence of gaming disorder of 3.05% (confidence interval: [2.38, 3.91]) [3]. Another meta-analysis of 155 reports including 407,620 participants from 33 different countries found a pooled prevalence of Internet Gaming Disorder (IGD) of 9.9% (confidence interval: [8.6, 11.3]) among adolescents and young adults [4]. While some previous studies tend to refer interchangeably to the concepts IGD, cyber-dependence, Internet use disorder, problematic Internet use, and Internet addiction disorder, recent evidence has tended to differentiate and individualize IGD from other forms of problematic Internet use. Indeed, the different forms of online behavioral addictions (e.g., gaming, pornography, or gambling) have been found to result from distinct neurophysiological mechanisms [5, 6], and to emerge from different, specific motives than the addiction to the Internet itself [5, 7, 8]. These are the reasons why, in the fifth revision of the Diagnostic and Statistical Manual of Mental Disorders (DSM-5) in 2013 IGD has been recognized as a condition warranting further empirical study [9]; and in the 11th Revision of the International Classification of Diseases (ICD-11) in 2018, it was included by the World Health Organization as a mental disorder [10]. In the DSM-5, IGD encompasses 9 clinical symptoms (mixing five substance use and four gambling symptoms). In the ICD-11 formulation, gaming disorder refers to a pattern of persistent or recurrent gaming behavior manifested by only three core substance-like symptoms based on a unitary substance use approach (i.e., increasing priority given to gaming, impaired control over gaming, and escalation or continuation of gaming despite occurring negative consequences) [11]. Both the DSM-5 and ICD-11 pay attention to the condition of players in the past year, and include distress or clinically significant impairment for a clinical diagnosis. However, lower detection rates of gaming were reported when using ICD-11 criteria than DSM-5 criteria [11].

Recently, greater research interest has been devoted to understanding the effects of IGD on mental health [12, 13], and new standardized instruments assessing IGD in line with the DSM-5 criteria have been developed (e.g., [14]). However, the relationship between IGD and psychiatric disorders in general [15], as well as between IGD and psychosis in particular [16], remains poorly understood and largely under-researched. As for GD and psychosis, a scoping review published in 2022 also demonstrated that the existing literature is scarce [16]. Most of the previous evidence was based on case reports and case series describing psychotic episodes that could have been triggered by either excessive video gaming or sudden game play withdrawal [16]. The current study focused on how IGD may be linked to the development of psychotic experiences (PEs).

### The relationship between IGD and PEs

PEs refer to perceptual abnormalities and delusional beliefs occurring in healthy individuals with no history of diagnosed psychotic disorders [17–19], which interfere with functioning, are potentially distressing, [20, 21], confer increased risk of developing later full-blown psychosis [22], as well as other psychopathology and behavioral problems [23, 24]. In the present study, we refer to “psychoticism” and “paranoid ideations” as PEs. These constructs were originally designed to reflect a continuum of psychotic manifestations ranging from mild psychotic symptoms to florid psychosis [25]. Due to their definition and item content, the scales “paranoid ideation” and “psychoticism” were traditionally considered as a measure of “psychosis proneness” (e.g., [26–28]). For instance, previous research showed that the psychoticism scale was able to distinguish patients with schizophrenia from other patients [29]. Items of the psychoticism dimension (e.g., thought-broadcasting, thought-intrusion, auditory hallucinations, delusions of control) were designated to as “schizophrenia nuclear symptoms” [30].

Many explanations could be offered for the relationship between IGD and PEs. Both PEs and IGD have shown significant associations with social anxiety disorders [31, 32], social isolation, anhedonia, loneliness [33–35], substance use [36, 37], and other addictive behaviors [38, 39]. Also, IGD and psychosis share underlying biological, psychological, or environmental mechanisms, such as childhood maltreatment, bullying, and dysfunctional family environments [4, 40, 41], that may increase vulnerability to both pathologies. In addition, multiple brain functioning modifications due to excessive gaming may trigger an increased dopamine release [42, 43], and other neurobiological alterations (such as decreased activation in areas involved in inhibitory control, impaired executive function, cognitive control, and motivation [44–46]); which could, in turn, increase psychosis proneness in genetically predisposed individuals. In addition, numerous emotional and behavioral disturbances related to IGD may possibly increase vulnerability to psychosis, such as sleep deprivation [47, 48], depression, anxiety, stress, and bullying [4, 49]. To summarize, little is known about the underlying mechanisms that may moderate or mediate the relationship between cyberbullying and psychosis. In the next section, we expose the theoretically and empirically based literature supporting the possible mediating role of three variables, i.e., insomnia, distress and cyberbullying, in the association between IGD and PEs.

### Insomnia and cyberbullying as hypothesized mediators in the relationship between IGD and PEs

Previous studies provide some evidence for a possible association between IGD and a range of sleep problems [47]. In particular, pathological online gaming was found to be linked to decreased night sleep duration [50], reduced time spent in bed and late bedtimes [51], difficulties in falling asleep [52], decreased slow-wave sleep [53], delayed sleep phase syndrome, and insomnia [54]. Online gaming addicts often play with other gamers from different time zones [55]; they avoid logging off to minimize any losses and refrain from sleeping to gain more time in gaming [47, 56–58]. A Lebanese study found that high-school students with IGD has an average number of hours of sleep of 4.9 per night, and that 42.9% of these gamers reported waking up throughout the night to continue gaming online [50]. A study in Singapore found that heavy online gamers displayed a prevalence of insomnia symptoms of 28% [57]. On the other hand, insomnia has been demonstrated to increase the risk for developing mental health problems, including psychosis [59]. Insomnia is highly prevalent in individuals with psychotic disorders in different clinical stages of the disease [48, 60, 61]. Insomnia has been suggested to contribute to persistent or exacerbated psychotic symptoms over time [62–65], and even lead to emergence of de novo psychotic experiences in healthy individuals [66]. For instance, a large cross-country study involving 261,547 participants showed that having sleep problems led to increased odds for at least one psychotic symptom, even after adjusting for depression and anxiety [67]. Overall, sleep problems have been identified as a significant vulnerability factor in the development and persistence of young people’s psychotic symptoms and experiences [48, 68, 69].

Playing online video games has been associated with multiple online risks, including cyberbullying. Cyberbullying is defined as ‘willful and repeated harm inflicted through the medium of electronic text’ [70]. There is evidence that cyberbullying is a highly prevalent problem in the adolescent and young adult population [71]. Even though cyberbullying within online gaming communities remains an under-researched problem, sufficient evidence exists to support that increased use of online gaming is associated with greater involvement in cyberbullying [72–75], which is mainly due to the toxic culture widespread in some gaming communities [76, 77]. Cyberbullying behaviors include harassment for not performing well, ruining the experience for others, cheating, sabotage of games, and threatening violence; and have been found to persist in spite of game creators’ efforts to overcome them [78]. On the other hand, a growing body of research found that involvement in cyberbullying either as a bully or as a victim contributes to poor mental health [79–82], and was found to significantly correlate with psychosis in both clinical and non-clinical populations. For instance, a study including fifty individuals with attenuated psychotic symptoms found that 38% of the sample reported cyberbullying, mostly through text messages, instant messaging and Facebook [83]. In this study, cyberbullying was evaluated by asking respondents if they had been bullied or harassed through communication devices or technology [83]. A Turkish cross-sectional study found that engaging in cyberbullying (as assessed using self-developed questions) was significantly correlated with more severe psychoticism symptoms among undergraduate university students [84]. More recently, involvement in cyberbullying either as victims or as bullies has been demonstrated to be significantly associated with PEs in a sample of Chinese high school students [85].

### Rationale of the present study

**Fig. 1:**
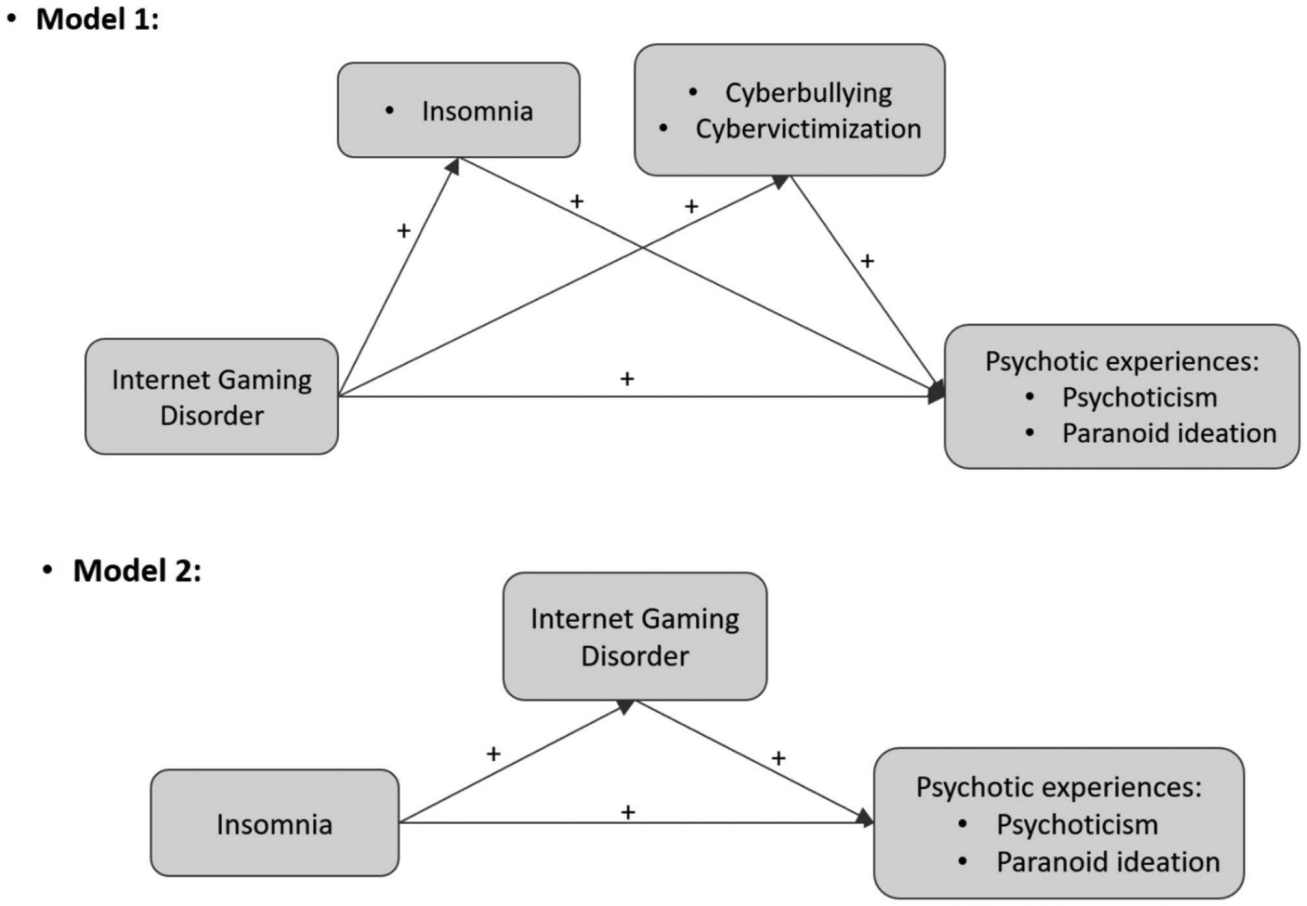
Conceptual models for the possible pathways linking IGD, insomnia, cyberbullying/cyber-victimization and psychotic experiences

Overall, the nature of the relationship between IGD and psychosis is unclear so far. In light of the above-mentioned observations, greater time spent in playing video games may expose to both insomnia and more toxic online environment with widespread cyberbullying. These two possible consequences of IGD may, in turn, be associated with greater PEs. Based on this theoretical framework, the present study proposes to fill some knowledge gaps in the existing literature. First, no studies have examined the indirect effects of cyberbullying and insomnia in the relationship between IGD and PEs, to the best of our knowledge. Investigating the role of these mediators may help elucidate potential mechanisms underlying the relationship between IGD and psychosis along the extended psychosis continuum. Second, previous studies have concentrated on investigating the interplay between PEs and Internet addiction or problematic Internet use in general [86–88]. A literature review published in 2018 [89] could find only one study (i.e., [90]) that specifically investigated the association between IGD and psychoticism. However, online gaming is a specific form of Internet usage that has its peculiarities compared to all other online activities [7]; and IGD was demonstrated to be a “conceptually different behavior” and a separate nosological entity than internet use disorder [91, 92]. Third, most of the previous research primarily focused on demonstrating a positive correlation between traditional bullying and PEs [93–96]. However, many studies have suggested that cyberbullying and traditional bullying are not the same [97]; with cyber forms of bullying being proposed to pose greater threats to psychosocial adaptation and cause worse effects on mental health compared to traditional bullying [98–100]. To contribute the body of the knowledge in this area, and based on the above-detailed literature, we propose a hypothesized model testing the possible indirect effects of insomnia severity, cyber-victimization and cyberbullying in the cross-sectional association between IGD and PEs in a sample of Tunisian university students. Because of the cross-sectional nature of the study, and given some previous evidence on a bidirectional causal relationship between IGD and insomnia [101], we propose to test two possible models (Fig. 1): IGD may precede both insomnia and cyberbullying (model 1), or insomnia may precede IGD (model 2). In addition, we decided to adjust for psychological distress in the models, as this variable showed established connections with insomnia [102], IGD [103], cyberbullying/cyber-victimization [104] and PEs [105].

## Methods

### Sample and procedure

We conducted a cross-sectional study over 4 months (February-May 2022). A non-probability convenience sampling technique was used for the study. Tunisian university students were approached at the end of their scheduled course classes, and invited by the researchers to participate if they were: (1) aged over 18 years, (2) affiliated to a public Tunisian university, (3) had no personal history of psychosis or antipsychotic medication intake, and (4) willing to participate. All students who met the eligibility criteria were self-administered a paper-and-pencil anonymous questionnaire. A total of 983 students were approached and accepted to participate. Students who reported any personal history of diagnosed mental disorders (n = 49) and those who did not complete the questionnaire (n = 83) were excluded, resulting in a total final sample of 851 students. All participants were provided full information on the study, and provided their informed consent to participate. The research protocol was approved by the authorities of each University, and by the ethics committee of the Razi psychiatric hospital, Manouba, Tunisia. The study was performed following the standards for medical research involving human subjects recommended by the Declaration of Helsinki for human research.

### Measures

The questionnaire comprised items about demographic information and four self-report measurement instruments (The Brief Symptom Inventory [BSI], the Internet Gaming disorder-20 Test [IGD-20], the Insomnia Severity Index [ISI], and the Revised Cyber Bullying Inventory–II [RCBI-II], and the Depression, Anxiety and Stress Scales [DASS-21]). The demographic information section included age, gender, family monthly income, housing area, substance use (cigarettes, alcohol, cannabis, other illegal drugs), family history of psychiatric illness, owing a smartphone (Yes/No), making money from gaming (Yes/No), difficulty connecting to the internet (Yes/No), average time spent on the internet daily (in minutes), and average Time spent on playing video games per day (in minutes).

#### The BSI

The BSI is a short form of the Symptom Checklist Revised (SCL-90-R) [25, 106], and consists of 53 items divided into nine symptom dimensions. The 6-item paranoid ideation subscale and the 10-item psychoticism subscale of the BSI [107, 108] were used to measure participants’ PEs. The “paranoid ideation” and “psychoticism” dimensions have been largely used to assess PEs in the general population (e.g., [30, 109–116]), and have been included as measurement tools of PEs in previous meta-analyses (e.g., [117, 118]). The paranoid ideation subscale detects paranoia symptoms that involve delusion of reference and suspicion; while the psychoticism subscale identifies psychotic symptoms such as auditory hallucination, experience of thought insertion, thought control, and thought being revealed. Each item evaluates to what extent respondents were bothered by a given symptom during the past week, and was rated on a five-point Likert scale, ranging from 0 (“not at all”) to 4 (“extremely”). The separate scores of the two subscales were used in analyses, with higher scores indicating greater severity of symptoms on the corresponding dimension. The Arabic version of the BSI used in the present study has shown good validity and reliability (Cronbach α = 0.73 and 0.83 for the Paranoid Ideation and Psychoticism subscales, respectively) [119], and has previously been used in Arab student populations (e.g., [120]). The Arabic psychoticism and paranoid ideation subscales yielded α values of 0.81 and 0.77, respectively.

#### The IGD-20

This is a 20-item scale designed by Pontes et al. in 2014 [121] to measure IGD according to the nine DSM-5 criteria [122], and following the Griffith’ components model of addiction [123]. This measure was chosen because its Arabic version showed very good psychometric properties [124]. Respondents are requested to answer questions asking about their offline and online video gaming behaviors within the previous 12 months. Items are rated on a five-point Likert-type scale, ranging from 1 (strongly disagree) to 5 (strongly agree). Respondents who scored between 50 and 70 on the IGD-20 were considered as being currently at risk of IGD, and those scoring 71 or more were considered as having IGD [14, 125]. Our sample yielded an α Cronbach for the total IGD-20 scores of 0.926.

#### The ISI

The ISI is the most widely used measure of insomnia. It is a 7-item measure asking about sleep maintenance, early morning awakening problems, severity of sleep onset, noticeability of sleep problems by others, interference of sleep difficulties with daytime functioning, sleep dissatisfaction, and distress caused by the sleep difficulties [126]. The ISI has been found reliable for assessing the severity nature, and impact of insomnia symptoms [126]. The Arabic validated version of the ISI was used in this study [127]. The Cronbach’s α value was 0.78 for total scores in our sample.

#### The RCBI-II

This scale is composed of 20 items and two subscales to indicate either the listed cyberbullying behavior was done by the respondent as a cyberbully, or happened to them as a cyber-victim (cyber-victimization, 10 items; cyberbullying, 10-items) [128]. Items are scored on a four-point scale that ranges from 1 (never) to 4 (more than three times). Higher scores refer to greater cyberbullying/cyber-victimization experiences. The two above-mentioned subscales have been used separately in analyses. To the best of our knowledge, the RCBI-II is the only cyberbullying measure validated in the Arabic language [129], which revealed Cronbach’s α coefficients for the cyber-victimization and the cyberbullying subscales of 0.85 and 0.80, respectively. In our study, alpha values were calculated as 0.81 for the cyber-victimization scale, and as 0.79 for the cyberbullying subscale.

#### The DASS-21

This measure is composed of a 21-item assessing the severity of psychological distress symptoms through three subscales: DASS-depression (7 items), DASS-anxiety (7 items), and DASS-stress (7 items) [130]. Each item is scored on a four-point Likert-type scale (from “I strongly disagree” =0 to “I totally agree” =3). Greater scores indicate higher distress levels. The Arabic DASS-21 was used [131]; it showed good reliability in the present sample, with Cronbach’s alpha values of 0.79 for depression, 0.76 for anxiety and 0.81 for stress.

### Statistical analysis

SPSS software version 23 was used to conduct data analysis. We had no missing data in our database. McDonald’s omega values were recorded for reliability analysis of all scales and subscales. The paranoid ideation and psychoticism scores were normally distributed; therefore, the Student t test was used to compare two means, ANOVA test to compare three or more means, and the Pearson test to compare two continuous variables. Bonferroni correction was done to take into account the multiple analyses; the corrected *p* value was estimated at 0.003 and was calculated by dividing 0.05 by the total number of variables included in the analysis (= 19). To check for a significant indirect effect of insomnia severity, cyberbullying and cyber-victimization between IGD and paranoid ideation/psychoticism, we conducted a mediation analysis using SPSS PROCESS v3.4 model 4 with three pathways; pathway A from the independent variable to the mediator, pathway B from the mediator to the dependent variable and pathway C from the independent to the dependent variable. Variables that showed a significant corrected *p* value in the bivariate analysis were entered in the mediation analysis. Significance was set at a p < 0.05.

## Results

A total of 851 participants enrolled in this study, with a mean age of 21.26 ± 1.68 and 53.7% females. All sociodemographic and other characteristics of the participants are summarized in Table 1. According to the above-mentioned cutoff values, 25% of students were at risk of IGD, and 1.8% had an IGD.

**Table 1:** Sociodemographic and other characteristics of the participants (N = 851)

Variable	n (%)
Gender	
Male	394 (46.3%)
Female	457 (53.7%)
Family monthly income	
<500	55 (6.5%)
500–1000	221 (26.0%)
1000–2000	320 (37.6%)
2000–3000	141 (16.6%)
>3000	114 (13.4%)
Housing area	
Urban	743 (87.3%)
Rural	108 (12.7%)
Cigarettes smoking	
No	620 (72.9%)
Yes	231 (27.1%)
Alcohol drinking	
No	690 (81.1%)
Yes	161 (18.9%)
Cannabis use	
No	768 (90.2%)
Yes	83 (9.8%)
Other illegal drug use	
No	828 (97.3%)
Yes	23 (2.7%)
Family history of psychiatric illness	
No	791 (92.9%)
Yes	60 (7.1%)
Owing a smartphone	
No	14 (1.6%)
Yes	837 (98.4%)
Making money from gaming	
No	834 (98.0%)
Yes	17 (2.0%)
Difficulty connecting to the internet	
No	714 (83.9%)
Yes	137 (16.1%)
	**Mean ± SD**
Age (in years)	21.26 ± 1.68
Time spent on the internet per day (in minutes)	5.11 ± 3.31
Time spent on gaming (in minutes)	61.90 ± 94.64
Internet gaming disorder (IGD-20 total scores)	41.70 ± 13.55
Insomnia (ISI total scores)	11.30 ± 5.38
Paranoid ideation (BSI)	8.61 ± 4.75
Psychoticism (BSI)	10.38 ± 6.52
Cyberbullying (RCBI-II)	13.31 ± 4.21
Cyber-victimization (RCBI-II)	14.92 ± 5.00

IGD-20: The Internet Gaming disorder-20 Test; ISI: Insomnia severity index; BSI: The Brief Symptom Inventory; RCBI-II: the Revised Cyber Bullying Inventory–II

### Bivariate analysis

The results of the bivariate analysis are summarized in Tables 2 and 3. Higher psychoticism mean scores were found in smokers compared to nonsmokers. Having a family history of psychiatric disorders was significantly associated with higher paranoid ideation and psychoticism. Higher IGD, insomnia severity, cyberbullying and cyber-victimization, time spent on the internet per day and time spent on gaming were significantly associated with more paranoid ideation and psychoticism.

**Table 2:** Bivariate analysis of factors associated with paranoid ideation and psychoticism

Variable	Paranoid ideation	*p*	Psychoticism	*p*
Gender		0.098		0.964
Male	8.31 ± 4.90		10.39 ± 6.32	
Female	8.86 ± 4.61		10.37 ± 6.69	
Family monthly income		0.564		0.181
<500	9.00 ± 4.74		10.93 ± 6.89	
500–1000	8.52 ± 4.44		10.21 ± 6.33	
1000–2000	8.79 ± 4.90		10.78 ± 6.44	
2000–3000	8.68 ± 4.73		10.57 ± 6.45	
>3000	7.97 ± 4.96		9.11 ± 6.91	
Housing area		0.120		0.381
Urban	8.62 ± 4.77		10.46 ± 6.56	
Rural	8.50 ± 4.67		9.87 ± 6.21	
Cigarettes smoking		0.111		**0.001**
No	8.45 ± 4.70		9.92 ± 6.26	
Yes	9.03 ± 4.88		11.61 ± 7.02	
Alcohol drinking		0.005		0.146
No	8.38 ± 4.75		10.23 ± 6.54	
Yes	9.55 ± 4.67		11.06 ± 6.37	
Cannabis use		0.110		0.091
No	8.52 ± 4.75		10.26 ± 6.53	
Yes	9.40 ± 4.74		11.53 ± 6.33	
Other illegal drug use		0.531		0.111
No	8.59 ± 4.75		10.32 ± 6.49	
Yes	9.22 ± 4.70		12.52 ± 7.36	
Family history of psychiatric illness		**0.001**		**0.001**
No	8.45 ± 4.73		10.18 ± 6.44	
Yes	10.60 ± 4.63		13.05 ± 7.03	
Making money from gaming		0.192		0.267
No	8.64 ± 4.74		10.42 ± 6.52	
Yes	7.12 ± 5.16		8.65 ± 6.10	
Difficulty connecting to the internet		0.455		0.221
No	8.55 ± 4.74		10.26 ± 6.61	
Yes	8.88 ± 4.82		11.01 ± 5.97	

Numbers in bold indicate significant *p* values after Bonferroni correction

**Table 3:** Correlation matrix of continuous variables

	1	2	3	4	5	6	7	8	9	10	11
1. Paranoid ideation	1										
2. Psychoticism	**0.57**	1									
3. Internet gaming disorder	**0.20**	**0.29**	1								
4. Insomnia severity	**0.35**	**0.35**	**0.23**	1							
5. Cyberbullying perpetration	**0.21**	**0.27**	**0.24**	**0.12**	1						
6. Cyberbullying victimization	**0.19**	**0.28**	**0.13**	**0.20**	**0.44**	1					
7. Depression	**0.51**	**0.59**	**0.11**	**0.43**	**0.17**	**0.23**	1				
8. Anxiety	**0.47**	**0.57**	0.09	**0.39**	**0.16**	**0.30**	**0.68**	1			
9. Stress	**0.56**	**0.57**	0.09	**0.47**	**0.19**	**0.26**	**0.74**	**0.74**	1		
10. Age	− 0.04	− 0.08	− 0.07	0.01	− 0.04	− 0.06	− 0.05	− 0.05	0.01	1	
11. Time spent on the internet per day	**0.21**	**0.19**	**0.16**	**0.22**	**0.10**	**0.13**	**0.16**	**0.19**	**0.21**	− 0.03	1
12. Time spent on gaming	**0.18**	**0.19**	**0.45**	**0.18**	**0.20**	0.09	0.05	0.05	0.08	0.02	**0.36**

Numbers in bold indicate significant *p* values after Bonferroni correction

### Mediation analysis

**Fig. 2:**
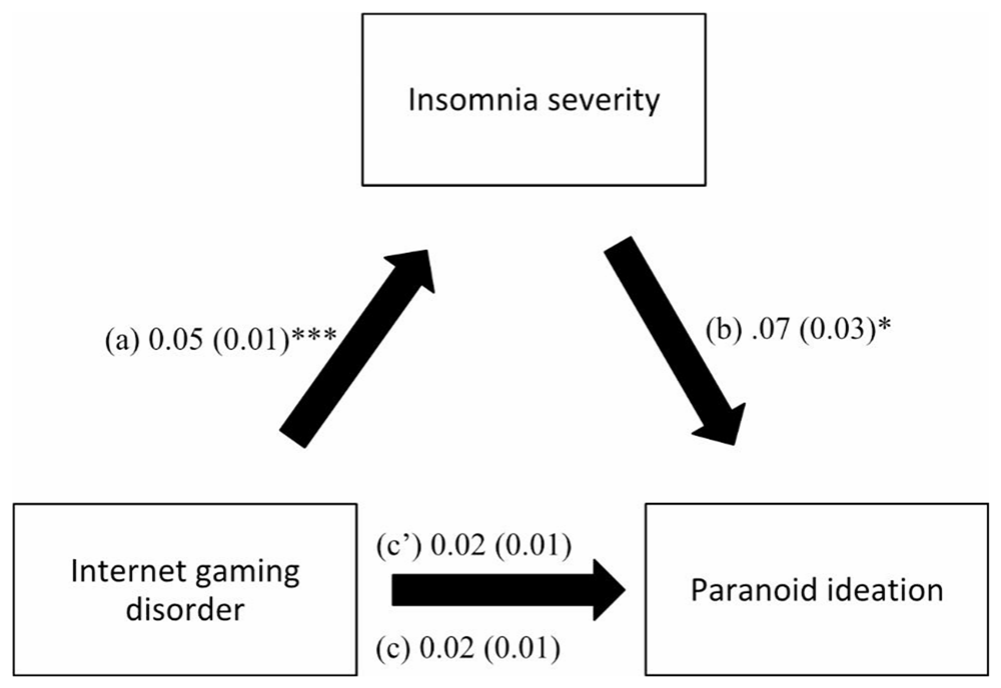
(**a**) Relation between internet gaming disorder and insomnia severity (R^2^ = .247); (**b**) Relation between insomnia severity and paranoid ideation (R^2^ = .358); (**c**) Total effect of internet gaming disorder on paranoid ideation (R^2^ = .354); (**c’**) Direct effect of internet gaming disorder on paranoid ideation. Numbers are displayed as regression coefficients (standard error). *p < 0.05; ***p < 0.001

The results of the mediation analysis showed that insomnia severity fully mediated the association between IGD and paranoid ideation (Table 4). Higher IGD was significantly associated with more insomnia severity, which was, in turn, significantly associated with more paranoid ideation (Fig. 2).

**Table 4:** Mediation analyses results, taking the internet gaming disorder as the independent variable, insomnia severity/cyber-victimization/cyberbullying as mediators and paranoid ideation/psychoticism as dependent variables

	Direct effect			Indirect effect		
	Beta	SE	*P*	Beta	Boot SE	Boot CI
**Model 1: Paranoid ideation as the dependent variable.**						
Insomnia severity	0.02	0.01	0.112	0.004	0.002	0.0003; 0.008*
Cyberbullying	0.02	0.01	0.112	0.003	0.002	− 0.0002; 0.007
Cyber-victimization	0.02	0.01	0.112	< 0.001	0.0001	− 0.001; 0.001
**Model 2: Psychoticism as the dependent variable.**						
Insomnia severity	0.07	0.01	< 0.001	0.003	0.002	− 0.001; 0.008
Cyberbullying	0.07	0.01	< 0.001	0.005	0.002	0.001; 0.010*
Cyber-victimization	0.07	0.01	< 0.001	− 0.0001	0.001	− 0.003; 0.002

Model 1: Family history of psychiatric illness, time spent on the internet per day, time spent on gaming, depress, anxiety and stress

Model 2: Cigarette smoking, Family history of psychiatric illness, time spent on the internet per day, time spent on gaming, depress, anxiety and stress

*indicates significant mediation; results were adjusted over variables that showed a significant *p* value after Bonferroni correction

**Fig. 3:**
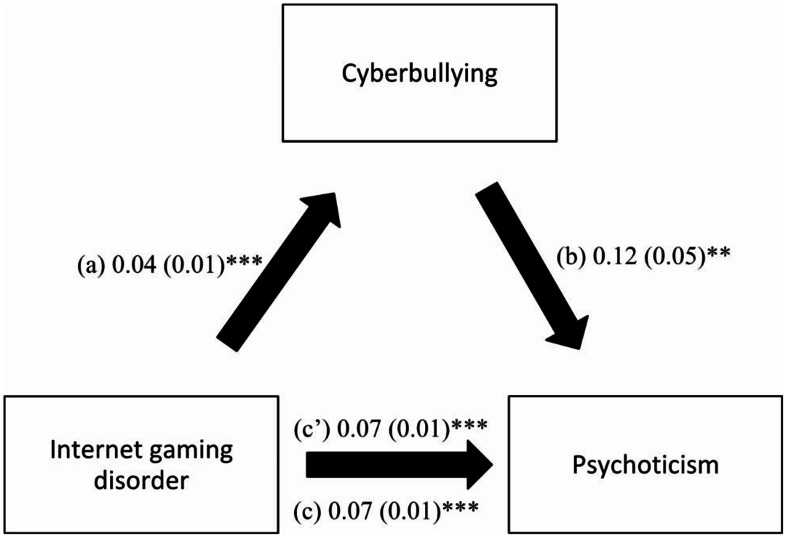
(**a**) Relation between internet gaming disorder and cyberbullying (R^2^ = .260); (**b**) Relation between cyberbullying and psychoticism (R^2^ = .452); (**c**) Total effect of internet gaming disorder on psychoticism (R^2^ = .447); (**c’**) Direct effect of internet gaming disorder on psychoticism. Numbers are displayed as regression coefficients (standard error). **p < 0.01; ***p < 0.001

Cyberbullying mediated the association between IGD and psychoticism (Table 4). Higher IGD was significantly associated with more cyberbullying, which was, in turn, significantly associated with more psychoticism. Finally, higher IGD was significantly and directly associated with higher psychoticism. Thus, IGD has both direct and indirect effects on psychoticism via cyberbullying (Fig. 3).

**Fig. 4:**
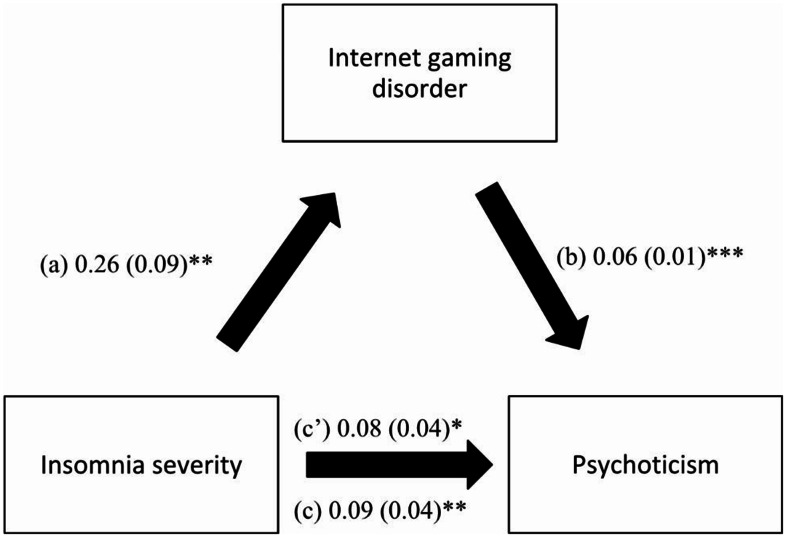
(**a**) Relation between insomnia severity and internet gaming disorder (R^2^ = .252); (**b**) Relation between internet gaming disorder and psychoticism (R^2^ = .455); (**c**) Total effect of insomnia severity on psychoticism (R^2^ = .444); (**c’**) Direct effect of insomnia severity on psychoticism. Numbers are displayed as regression coefficients (standard error). *p < 0.05; **p < 0.01; ***p < 0.001

The results of the mediating effect of IGD between insomnia severity and paranoid ideation did not show a significant mediation (indirect effect: Beta = 0.004, Boot SE = 0.004, Boot CI − 0.002, 0.012). This model was adjusted over the following variables: Family history of psychiatric illness, time spent on the internet per day, time spent on gaming, depression, anxiety and stress, cyberbullying perpetration and cyberbullying victimization. However, IGD mediated the association between insomnia severity and psychoticism (indirect effect: Beta = 0.02, Boot SE = 0.01, Boot CI 0.004, 0.03). Higher insomnia severity was significantly associated with higher IGD; higher IGD was significantly associated with higher psychoticism, whereas higher insomnia severity was significantly associated with higher psychoticism (Fig. 4). This model was adjusted over the following variables: Family history of psychiatric illness, smoking, time spent on the internet per day, time spent on gaming, depression, anxiety and stress, cyberbullying perpetration and cyberbullying victimization.

## Discussion

In this study, we proposed to explore the mediating role of cyberbullying and insomnia in the paths leading from IGD to PEs. As our study is cross-sectional in nature, we also proposed to test the mediating role of IGD in the paths leading from insomnia to PEs. Our analyses indicated that the link between IGD and paranoid ideation is fully mediated through insomnia severity, and that the IGD-psychoticism relationship is partly mediated by cyberbullying. Additionally, we found that IGD acted as a mediator in the association between insomnia and psychoticism.

Our findings provided evidence to support a possible linkage between IGD and PEs. The assumption that IGD may be associated with PEs has been fueled by a more general and well-established relationship between Internet addiction and psychotic symptoms across the continuum [87, 88, 132–141]. To date, there has been a dearth of literature examining the relationship between IGD in particular, and PEs [16]. A few studies suggested that excessive playing of online games may be a trigger for new-onset psychotic symptoms (e.g., [142–144]). Consistent with our findings, a recent study found that Austrian and German adolescents and young adults (aged 14 to 30 years) who experienced high levels of PEs had an increased likelihood of reaching the cut-off for IGD, suggesting “a close relation” between these two entities [145]. Our findings along with previous research preliminarily suggest that IGD may act as an environmental stressor that could increase risk for developing psychosis in constitutionally vulnerable individuals, according to the diathesis-stress model [146]. We are aware, however, that based on our cross-sectional data, causal conclusions cannot be made. We draw readers’ attention to the possible bidirectionality between IGD and PEs, with pathways leading from PEs to IGD having been supported in prior research. For instance, some previous studies proposed that individuals would turn to playing video games to cope with their psychotic symptoms [16, 136, 143]. Only a very few cross-sectional studies have explored the relationship between GD and PEs, which do not allow to know whether PE can be caused by IGD or vice versa [16]. Future longitudinal studies are still needed to identify the nature of this relationship.

One important finding of this study was that insomnia and cyberbullying appear to play a mediating role between IGD-paranoid ideation and IGD-psychoticism, respectively, after controlling for psychological distress (i.e. depression, anxiety and stress), as well as other potential statistically identified confounding factors (i.e. family history of psychiatric illness, time spent on the internet per day, time spent on gaming). In particular, findings suggest that the total effect of IGD on paranoid ideation is transmitted through insomnia, whereas cyberbullying was found to account for some, but not all, of the relationship between IGD and psychoticism. In other words, the association between IGD and paranoid ideation can be fully explained by the mediational mechanism of insomnia, meaning that IGD does not influence paranoid ideation by itself but by first influencing insomnia. However, the association between IGD and psychoticism can be only partially explained by cyberbullying, meaning that other potential factors could play a role as mechanisms behind the association between IGD and psychoticism. Although this study is the first to explore the role of these mediators specifically in the association between IGD and PE, findings are in agreement with the available literature on the positive correlations of IGD with insomnia [50, 54, 57] and cyberbullying [72–75] on the one hand, and the positive correlations of insomnia [66, 67] and cyberbullying [84, 85] with PE on the other hand. Our results were also broadly consistent with previous findings that insomnia has a mediating effect between IGD and other mental health problems (e.g., suicidal ideation [147]). The identification of insomnia and cyberbullying as possible mediators may contribute to a better understanding of the mechanisms underscoring the association between IGD and PE; and, thereby, to novel and effective strategies aimed at reducing PE and its possible detrimental effects, or preventing psychosis onset in potentially vulnerable individuals. The readers’ attention is drawn to the fact that some previous findings suggested a bidirectional causal relationship between IGD and insomnia [101]. Although insomnia is better accepted as a mediator on the association between IGD and psychological problems [148], there remains a possibility that insomnia precedes IGD (e.g., [101]) as demonstrated in the present results. Therefore, two mediation models tested in our sample may imply that young adults with insomnia may experience subsequent IGD before developing PEs, or that those with IGD may have resulting insomnia and, consequently, develop PEs. Altogether, it appears that longitudinal study designs should be considered in future research to validate these causal relationships.

### Study limitations

Our findings need to be interpreted while considering certain limitations. The first limitation lies to the cross-sectional design and self-report nature of the study. Future prospective studies using more objective assessments are required to overcome these issues (e.g., Including peer-reports when assessing cyberbullying [149]). The use of structured clinical interviews is also recommended for assessing IGD and PEs in future research. Another limitation consists of the inclusion of university students, which could limit the generalizability of our findings to the broader community sample of wider sociodemographic characteristics. In addition, the “psychoticism” and “paranoid ideations” scales of the BSI were used in the present study to assess PEs, due to the unavailability of other more specific self-report measures of this construct that are validated in the Arabic language at the start of the survey (e.g., the Arabic validated CAPE-42 [150] and PQ-B [151] were made available in 2023). Finally, both insomnia and cyberbullying only partially mediated the paths from IGD to PEs, which implies that other possible mediators (e.g., stress, suicidality, aggression) still need to be investigated to help further explain this relationship.

### Study implications

Our study is among the very few to shed light on the positive direct association between IGD and PEs in a sample of community adults. It is of note that IGD and PEs share several negative consequences that can be amplified by their simultaneous presence [16]. Therefore, more clinical and research attention should be paid to the coexistence and interaction of these two conditions, in order to provide information and offer effective early support to concerned individuals. Although causality cannot be assessed with our cross-sectional study, we preliminarily and cautiously suggest that IGD may be regarded as a possible stressor leading to onset or exacerbation of PEs, and call for future longitudinal studies to confirm our assumptions. Our study is also among the first to provide an overview of the mediating roles of two important factors, i.e. insomnia and cyberbullying, both being commonly represented in adolescents and young adults [152, 153]. Young adulthood also represents the peak age of onset for mental disorders [154], including IGD and PEs. Our findings suggest that insomnia and cyberbullying may be regarded as a potential target for youth mental health promotion, as well as community-focused prevention and early intervention in psychosis. Sleep deprivation should be prevented, assessed and treated in heavy gamers. Findings also suggest that IGD should be evaluated, prevented and managed individuals with insomnia. More particular attention should be devoted to the huge potential for engaging in cyberbullying among online gamers. In addition, as results indicated only a partial mediation effect of cyberbullying, the mechanisms underlying the association between IGD and psychoticism remain to be fully elucidated, and future studies still need to explore the role of other potential mediators in this relationship.

## Conclusion

This study highlighted the mediating role of insomnia severity and cyberbullying in the IGD-paranoid ideation and IGD-psychoticism connections, respectively. These results could help clinicians and researchers gain better insight into the processes relating IGD to PE; and might open up avenues for novel intervention possibilities. Furthermore, the present findings call attention to the need for a more detailed longitudinal research on the complex relationship between IGD and the different facets of PE, and for identifying other possible mediators and/or moderators of this relationship.

## Data Availability

The datasets generated and/or analyzed during the current study are not publicly available due to restrictions from the ethics committee but are available from the corresponding author on reasonable request.
